# Clinical manifestations, prevalence, and risk factors of asthenopia: a systematic review and meta-analysis

**DOI:** 10.7189/jogh.16.04053

**Published:** 2026-02-06

**Authors:** Fan Song, Yanjun Liu, Ziwei Zhao, Xianwen Shang, Yueye Wang, Mengying Lai, Mingguang He, Yanxian Chen

**Affiliations:** 1 The Hong Kong Polytechnic University, School of Optometry, Hong Kong, China; 2 The Hong Kong Polytechnic University, Research Centre for SHARP Vision (RCSV), Hong Kong, China; 3 The Center for Eye and Vision Research (CEVR), Hong Kong, China

## Abstract

**Background:**

This meta-analysis aims to determine the clinical manifestations, prevalence, and risk factors of asthenopia across diverse populations.

**Methods:**

We systematically searched PubMed up to April 2024 for studies published within the last five years on asthenopia, without language or design restrictions. Reference lists were also reviewed. The study quality was evaluated using the Newcastle-Ottawa Scale. A random-effects meta-analysis was conducted to calculate proportions, prevalence rates, odds ratios (ORs) and their 95% confidence intervals (CIs).

**Results:**

Overall, 63 studies were included. The pooled prevalence of asthenopia detected via questionnaires or symptom report was 51% (95% CI = 50%, 52%). Subgroup analyses showed high prevalence among digital device users (90%) and computer workers (77%). During the COVID-19 pandemic, prevalence rose among adults (39%–45%), university students (36%–57%), and school-aged children (45%–64%). The most frequent ocular symptoms were eye tiredness (65%, 95% CI = 46%, 84%), eye strain (47%, 95% CI = 37%, 58%), and burning/irritation (43%, 95% CI = 35%, 51%). Musculoskeletal symptoms, including neck pain (45%, 95% CI = 28%, 62%) and shoulder pain (30%, 95% CI = 12%, 48%) were also prevalent. Neuropsychological symptoms included headache (50%, 95% CI = 41%, 59%) and difficulty concentrating (44%, 95% CI = 32%, 56%). Risk factors included short sleep duration (OR = 1.28; 95% CI = 1.04, 1.57), prior eye disease (OR = 2.59; 95% CI = 1.43, 4.69), prolonged screen time (OR = 1.15; 95% CI = 1.09, 1.21), and ambient conditions like air conditioning use (OR = 23.02; 95% CI = 4.94, 107.18). Protective measures included anti-glare filters (OR = 0.34; 95% CI = 0.19, 0.64), regular breaks (OR = 0.21; 95% CI = 0.09, 0.51), and computer use knowledge (OR = 0.20; 95% CI = 0.13, 0.30).

**Conclusions:**

Asthenopia is prevalent across diverse populations, characterised by a wide range of symptoms and influenced by modifiable risk factors. Our findings support a unified definition to improve clinical recognition and offer preliminary evidence to help shape future research on preventive strategies.

**Registration:**

PROSPERO: CRD42024536841.

Asthenopia, also known as visual fatigue or digital eye strain (DES), is a common condition characterised by a range of symptoms following prolonged visual tasks, particularly those involving digital screens [1–3]. Its symptoms commonly include eye strain, dryness, blurred vision, redness, and headache, among others, which can significantly impair quality of life, work productivity, and academic performance [4–7]. With the increasing dependence on digital devices in both professional and personal contexts, asthenopia has become an emerging global public health concern. Quantifying its prevalence across diverse populations, synthesising its clinical manifestations, and identifying risk factors are essential for improving diagnostic accuracy and guiding effective prevention and management strategies in the digital age.

Although many studies have investigated the prevalence of asthenopia in specific populations such as students, children, office workers, and the general population, considerable variation exists in the diagnostic criteria and symptom assessment tools employed [6,8–11]. Consequently, the reported symptoms and prevalence estimates vary widely. Moreover, previous systematic reviews have typically focused on prevalence estimates within limited demographic groups, with few offering a comprehensive synthesis of its clinical manifestations or risk factors [1,3]. To date, there is no consensus on a standardised symptom profile or diagnostic framework for asthenopia.

This lack of integration limits comparability across studies and hampers the development of effective clinical guidelines. Given the heterogeneity in assessment methods and population characteristics, a broader synthesis is urgently needed to better understand the condition and help inform the development of future diagnostic criteria and targeted interventions.

This systematic review and meta-analysis aim to fill these gaps by providing an overview of the global prevalence of asthenopia, synthesising its clinical manifestations, and identifying risk factors. Through this comprehensive approach, we hope to contribute to the development of more consistent and reliable diagnostic frameworks and inform preventive strategies to better address the growing burden of asthenopia.

## METHODS

Our systematic review and meta-analysis followed a prospectively registered protocol (PROSPERO CRD42024536841; registered on 29 April 2024) to investigate the epidemiology of asthenopia. We specifically addressed the following questions:

(1) What are the common clinical manifestations associated with asthenopia?

(2) What is the prevalence of asthenopia in diverse populations?

(3) Which risk factors are significantly linked to asthenopia?

The findings were reported in accordance with the Preferred Reporting Items for Systematic Reviews and Meta-Analyses (PRISMA) 2020 guidelines (Table S1 in the **Online Supplementary Document**).

### Literature search

We conducted a scoping review including a systematic search of major database, PubMed, for studies published in the past five years up to 26 April 2024 describing asthenopia in different populations. We also checked for possible relative citations in their reference lists derived from Scholar after identifying all eligible studies. We conducted this search without any restrictions in language or study design. Details of the search strategies and databases searched are described (Table S2 in the **Online Supplementary Document**).

### Study selection

Two reviewers (FS and ZZ) independently screened studies in a two-stage process, first reviewing titles and abstracts, followed by a full-text assessment for eligibility. Two reviewers participated in the selection process, with disagreements resolved through discussion with a third reviewer (YC). Studies were excluded if the abstract was irrelevant to clinical manifestations, prevalence, or risk factors of asthenopia; the study design was unsuitable for obtaining relevant information; insufficient details were reported; asthenopia was not clearly defined; or the full article was unavailable.

Asthenopia was defined as a diagnosis based on either subjective questionnaires or the presence of any symptoms related to asthenopia. We included studies reporting the prevalence, clinical manifestations (symptoms), and risk factors of asthenopia across different populations. In addition to cross-sectional studies, we also included non-cross-sectional studies that provided relevant data.

### Study quality evaluation and data extraction

Two independent reviewers (FS and ZZ) assessed study quality using the Newcastle-Ottawa Scale (NOS), adapted for cross-sectional and non-cross-sectional designs [12,13]. The evaluation focused on selection (maximum 5 stars), comparability (maximum 2 stars), and outcome ascertainment bias (maximum 3 stars). Studies were categorised based on their total NOS score as follows: low risk of bias (high quality) if ≥7 stars, medium risk of bias (moderate quality) if 5–6 stars, and high risk of bias (low quality) if ≤4 stars. Data extraction was conducted independently by the two reviewers using a pre-piloted form.

### Statistical analysis

We conducted a meta-analysis using Stata, version 18.0 (StataCorp LLC, College Station, TX, USA), to estimate the pooled prevalence of asthenopia and clinical manifestations, reported as proportions with 95% confidence intervals (CIs), and risk factors associated with asthenopia, reported as odds ratios (ORs) with 95% CIs. For the prevalence estimates, we applied the Freeman-Tukey double arcsine transformation to stabilise variances prior to pooling the data. All analyses employed the DerSimonian and Laird random-effects model to account for between-study heterogeneity. Heterogeneity was quantified using the *I*^2^ statistic, with *I*^2^≥60% indicating substantial heterogeneity. Subgroup analyses were performed based on population types, including computer workers, digital device users, general adults, medical professionals, university students, school-aged children, and university staff. Additionally, within each subgroup, pooled prevalence estimates were calculated stratified by diagnostic method (*e.g*. single symptom, Computer Vision Syndrome Questionnaire (CVS-Q)), to explore the impact of different diagnostic criteria on reported prevalence. Sensitivity analyses included

(1) excluding studies with high risk of bias,

(2) re-estimating prevalence using a logit-transformed random-effects model, and

(3) restricting the analysis to studies that used validated questionnaires.

Forest plots were generated to visualise the prevalence, clinical manifestations, and risk factors estimates with 95% confidence intervals.

## RESULTS

After removing six duplicate or retracted records from the initial 3876 citations, a total of 3870 records were screened. Of these, 105 full-text articles were assessed for eligibility, and 63 studies were ultimately included in the systematic review (Figure S1 in the **Online Supplementary Document**). The included studies comprised 58 cross-sectional studies and five non-cross-sectional studies (one randomised controlled trial, three cohort studies, and one experimental study). All selected studies were conducted and published before April 2024.

### Characteristics of included studies

Among the 63 studies, the majority originated from India and Arabian countries (each contributing 11 studies, 17.5%) [9,10,14–33], followed by China (n = 5, 7.9%) [34–38], and Nepal (n = 4, 6.3%) [8,39–41]. Other contributing countries included Spain [42,43], Italy [7,44], Egypt [45,46], Jordan [47,48], Pakistan [49,50], Lebanon [6,51], Thailand [52,53], and African regions [54,55], each contributing two studies (3.2%). Single studies were conducted in Romania [56], Portugal [11], France [57], Greece [58], Korea [59], Japan [60], the USA [61], Australia [62], Ethiopia [63], Paraguay [64], Peru [65], Sudan [66], and Turkey [67] (each contributing one study, 1.6%). Additionally, three studies were multinational. Regarding diagnostic methods for asthenopia, 26 studies utilised the validated CVS-Q questionnaire [7,9,10,14,15,22,23,28,47,53–55,64,68–70], two used the Convergence Insufficiency Symptom Survey (CISS) [11,51], one used the Computer Vision Symptom Scale (CVSS17) [32], one employed the Asthenopia Survey Questionnaire-11 (ASQ-11) [34], and 18 relied on the presence of symptoms [6,16,19–21,26,27,31,35,37,39,41,45,63,66,67]. Clinical manifestations or symptoms of asthenopia were reported in 42 studies, while 50 studies reported its prevalence, and 24 studies examined risk factors.

### Quality of included studies

Overall, 66.7% (n/N = 42/63) of the cross-sectional or cohort studies assessed using the adapted Newcastle-Ottawa Scale (NOS) had a low risk of bias, while 25.4% (n/N = 16/63) had a medium risk of bias, and 7.9% (n/N = 5/63) had a high risk of bias (Table S3 in the **Online Supplementary Document**).

### Clinical manifestations of asthenopia

The most prevalent clinical manifestations of asthenopia were ocular symptoms. Eye tiredness was the most frequently reported symptom, with a pooled prevalence of 65% (95% CI = 0.46, 0.84; 7 studies; 1599 events), followed by eye strain or fatigue at 47% (95% CI = 0.37, 0.58, 19 studies; 6599 events). Burning or irritation was reported by 43% (95% CI = 0.35, 0.51; 33 studies; 8307 events), and itching was noted in 39% (95% CI = 0.28, 0.49; 19 studies; 4683 events), both representing significant symptoms. Lesser yet substantial occurrences included light sensitivity at 38% (95% CI = 0.31, 0.45; 19 studies; 3226 events) and eye pain at 37% (95% CI = 0.25, 0.49; 22 studies; 3819 events). Symptoms such as blurred vision and deteriorated vision appeared in 34% (95% CI = 0.19, 0.49; 34 studies; 5508 events) and 38% (95% CI = 0.26, 0.50; 10 studies; 1657 events) of cases, respectively. Eye dryness was reported by 35% (95% CI = 0.26, 0.44; 25 studies; 4927 events), and difficulty focusing or reading was observed in 34% (95% CI = 0.26, 0.43; 17 studies; 2637 events), indicating uncomfortable and inefficient vision.

Musculoskeletal symptoms, though less frequent than ocular symptoms, highlighted the physical strain associated with asthenopia. Neck pain had a pooled prevalence of 45% (95% CI = 0.28, 0.62; 11 studies; 1792 events), followed by shoulder pain at 30% (95% CI = 0.12, 0.48; 7 studies; 866 events), and back pain at 28% (95% CI = 0.13, 0.44; 6 studies; 658 events).

Neuropsychological symptoms were led by headache, which affected 50% (95% CI = 0.41, 0.59; 36 studies; 9543 events) of individuals, emphasising the link between visual strain and neurological discomfort. Difficulty in concentration showed a slightly lower prevalence at 44% (95% CI = 0.32, 0.56; three studies; 495 events). Nausea and dizziness, though less common at 29% (95% CI = 0.25, 0.34; one study; 126 events) and 11% (95% CI = −0.09, 0.31; two studies; 81 events), respectively, and memory or sleep disturbances, reported by 32% (95% CI = 0.18, 0.46; two studies; 174 events), suggested broader neuropsychological effects. Emotional distress, while low at 18% (95% CI = 0.08, 0.29; two studies; 98 events), highlighted the potential for visual strain to influence mental well-being.

**Figure 1:**
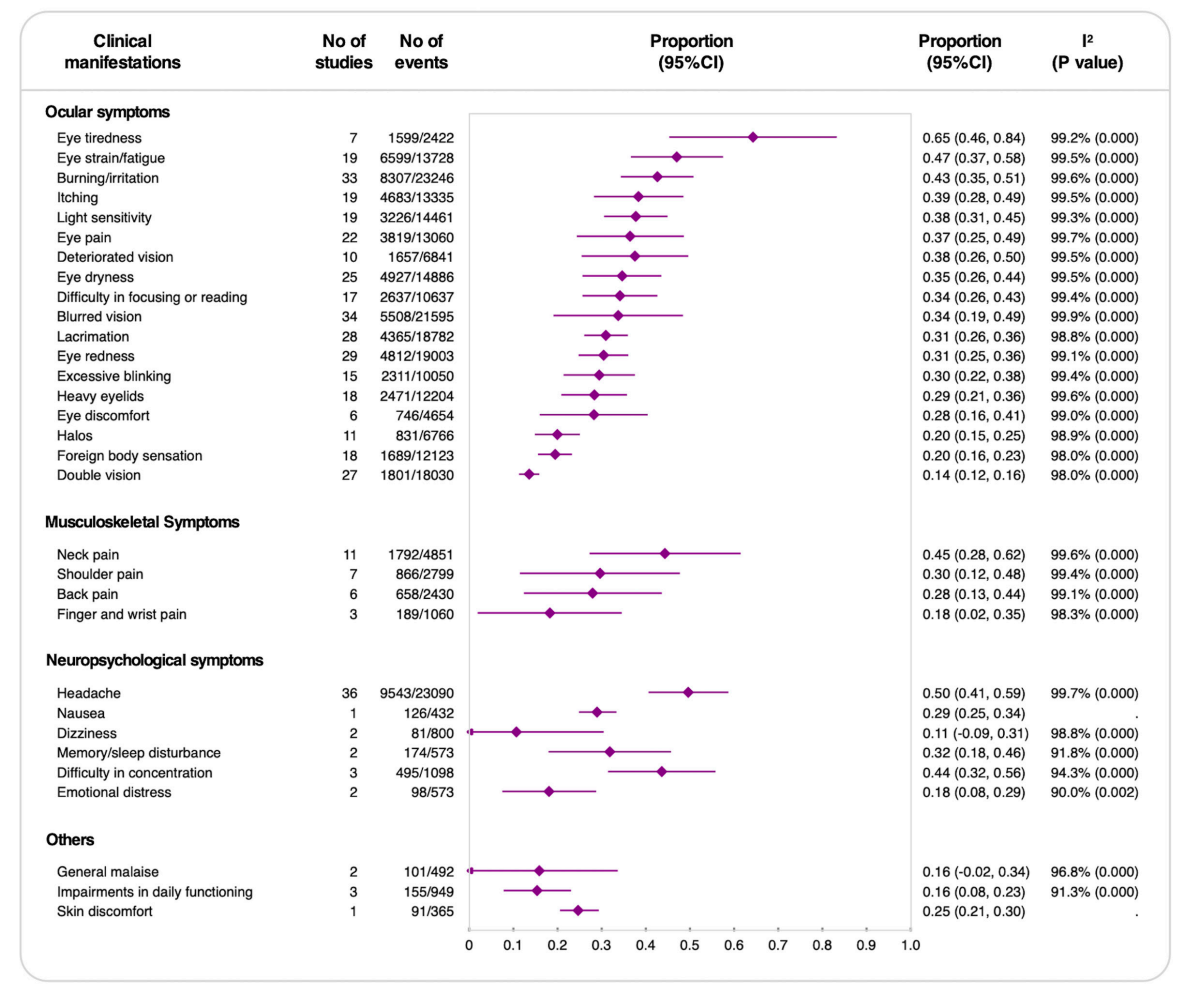
Rates of clinical manifestations of asthenopia. CI – confidence interval, *I*^2^ – inconsistency index (measure of between-study heterogeneity).

In addition, general malaise and impairments in daily function were reported in 16%, while skin discomfort had a slightly higher prevalence of 25% (95% CI = 0.21, 0.30; one study; 91 events). Detailed results of clinical manifestations are summarised (**Figure 1**).

### Prevalence of asthenopia

**Figure 2:**
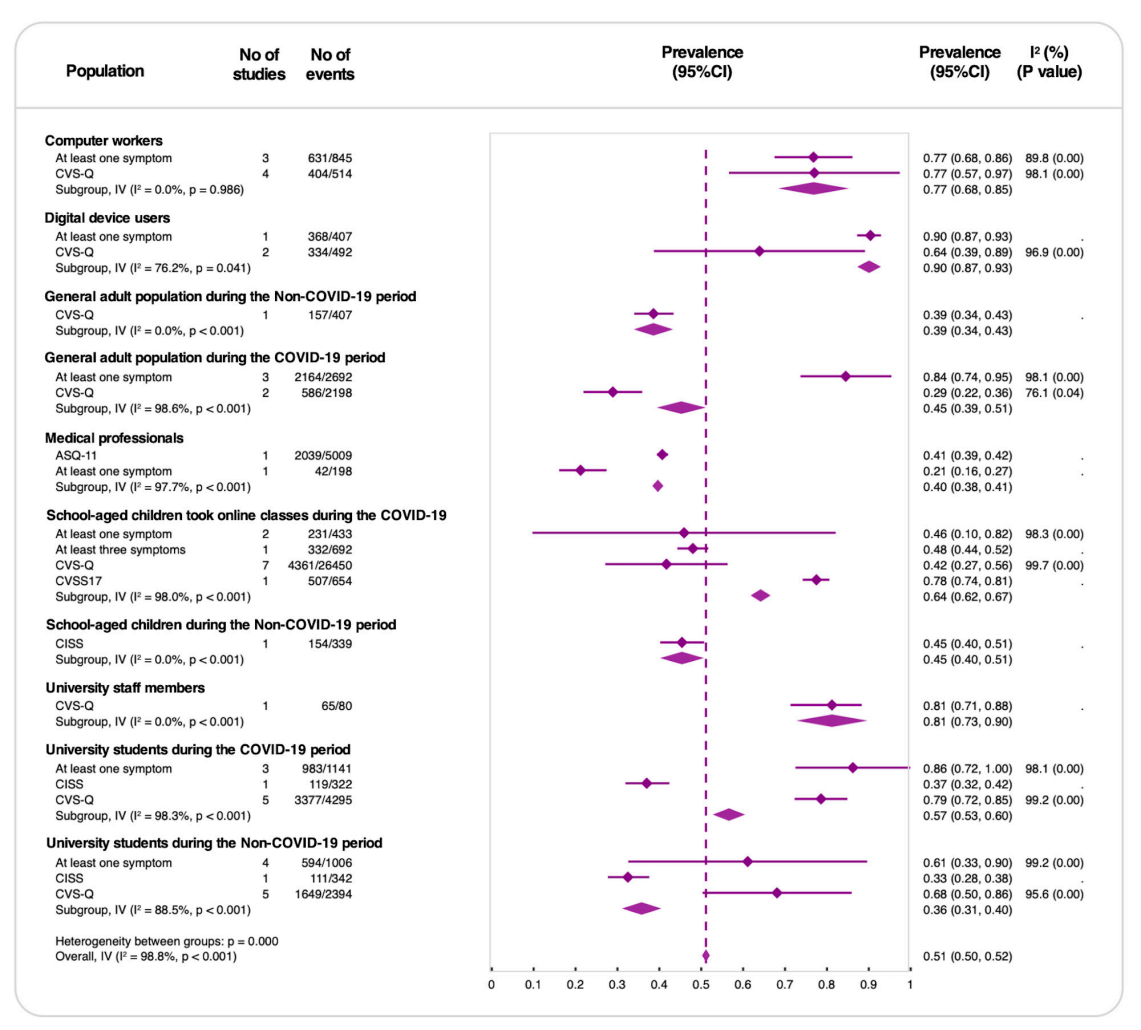
Prevalence of asthenopia by population type. ASQ-11 – Asthenopia Survey Questionnaire-11, CI – confidence interval, CISS – Convergence Insufficiency Symptom Survey, CVS-Q – Computer Vision Syndrome Questionnaire, CVSS17 – Computer Vision Symptom Scale, *I*^2^ – inconsistency index (measure of between-study heterogeneity).

The pooled prevalence of asthenopia across various populations was 51% (95% CI = 0.50, 0.52), indicating that more than half of the studied individuals experienced symptoms of asthenopia. However, significant heterogeneity was observed among subgroups (*I*^2^ = 98.8%, *P* < 0.001), suggesting that variations in population characteristics, diagnostic criteria, and study settings contribute to the differences in results (**Figure 2**).

Among specific population groups, computer workers had a prevalence of 77% confirmed by at least one symptom, which remained the same when assessed using the CVS-Q questionnaire. This group exhibited minimal heterogeneity (*I*^2^ = 0%, *P* = 0.986), indicating consistent findings. Digital device users showed a higher prevalence of 90% (95% CI = 0.87, 0.93) but with greater variability across studies (*I*^2^ = 76.2%, *P* = 0.041), suggesting that prevalence fluctuates depending on study conditions. In contrast, medical professionals had a lower pooled prevalence of 41% (95% CI = 0.39, 0.42) based on ASQ-11 or symptom-based assessments, accompanied by substantial heterogeneity (*I*^2^ = 97.7%, *P* < 0.001). University staff members demonstrated a prevalence of 81% (95% CI = 0.73, 0.90) according to a single CVS-Q-based study.

The COVID-19 pandemic significantly impacted asthenopia prevalence. In the general adult population, prevalence increased from 39% (95% CI = 0.34, 0.43) before the pandemic to 45% (95% CI = 0.39, 0.51) during the pandemic, reflecting lifestyle changes such as increased screen time due to remote work and social distancing measures. Similarly, prevalence among university students rose from 36% (95% CI = 0.31, 0.40) pre-pandemic to 57% (95% CI = 0.53, 0.60) during the pandemic. School-aged children also experienced an increase from 45% (95% CI = 0.40, 0.51) to 64% (95% CI = 0.62, 0.67), potentially influenced by shifts to online learning.

A sensitivity analysis restricted to studies with moderate or low risk of bias yielded a pooled prevalence of 60%, compared with 51% in the primary analysis including all studies (Figure S2 in the **Online Supplementary Document**). This suggests that the overall prevalence is somewhat influenced by study quality. In the logit-transformed random-effects sensitivity analysis, the overall pooled prevalence remained 51%; only minor numerical changes were observed, with the prevalence in the general population during the COVID-19 period increasing from 45 to 55% compared with the main analysis (Figure S3 in the **Online Supplementary Document**). When the analysis was restricted to studies using validated questionnaires, the overall pooled prevalence decreased from 51 to 46% (Figure S4 in the **Online Supplementary Document**).

### Risk factors of asthenopia

A total of 34 distinct risk factors for asthenopia were identified across the included studies and categorised into six classifications: demographic and lifestyle factors, vision and eyewear factors, digital device use and type, screen setup and ergonomics, ambient conditions, and protective measures. Among them, 22 factors were significantly associated with increased or decreased risk. Factors associated with an increased risk of asthenopia included short sleep duration (OR = 1.28; 95% CI = 1.04, 1.57; two studies; 26 975 participants), a history of eye disease (OR = 2.59; 95% CI = 1.43, 4.69; six studies; 2766 participants), and refractive error such as myopia (OR = 1.51; 95% CI = 1.27, 1.81; one study; 21 966 participants), hyperopia (OR = 1.56; 95% CI = 1.10, 2.30; one study; 3095 participants), and astigmatism (OR = 1.40; 95% CI = 1.06, 1.81; one study; 25 061 participants). Contact lens wear (OR = 1.33; 95% CI = 1.10, 1.62; four studies; 4646 participants), prolonged digital screen time per additional hour (OR = 1.15; 1.09, 1.21; 12 studies; 2614 participants), and poor screen ergonomics, including improper sitting posture (OR = 2.02; 95% CI = 1.51, 2.70; three studies; 23 126 participants) and non-adjustable screens (OR = 3.08; 95% CI = 1.70, 5.58; one study; 453 participants), were also identified as risk factors. In terms of ambient conditions, dark indoor illumination (OR = 4.89; 95% CI = 1.33, 17.98; one study; 365 participants), low indoor humidity (OR = 2.39; 95% CI = 1.06, 5.38; one study; 365 participants), air conditioner usage (OR = 23.02; 95% CI = 4.94, 107.18; one study; 108 participants), exposure to air pollution (OR = 5.67; 95% CI = 1.28, 25.16; one study; 108 participants), and exposure to windy environments (OR = 3.59, 95% CI = 1.24, 10.42; one study; 108 participants) significantly increased the likelihood of asthenopia.

In contrast, several protective factors were associated with a decreased risk of asthenopia, including good sleep quality (OR = 0.24; 95% CI = 0.20, 0.30; one study; 5009 participants), use of anti-glare filters (OR = 0.34; 95% CI = 0.19, 0.64; four studies; 1684 participants), exposure to natural light (OR = 0.01; 95% CI = 0.00, 0.08; one study; 455 participants), eye exercises (OR = 0.70; 95% CI = 0.65, 0.77; one study; 21 966 participants), taking regular breaks (OR = 0.21; 95% CI = 0.09, 0.51; eight studies; 25 866 participants), and having computer-use knowledge (OR = 0.20; 95% CI = 0.13, 0.30; three studies; 1043 participants).

**Figure 3:**
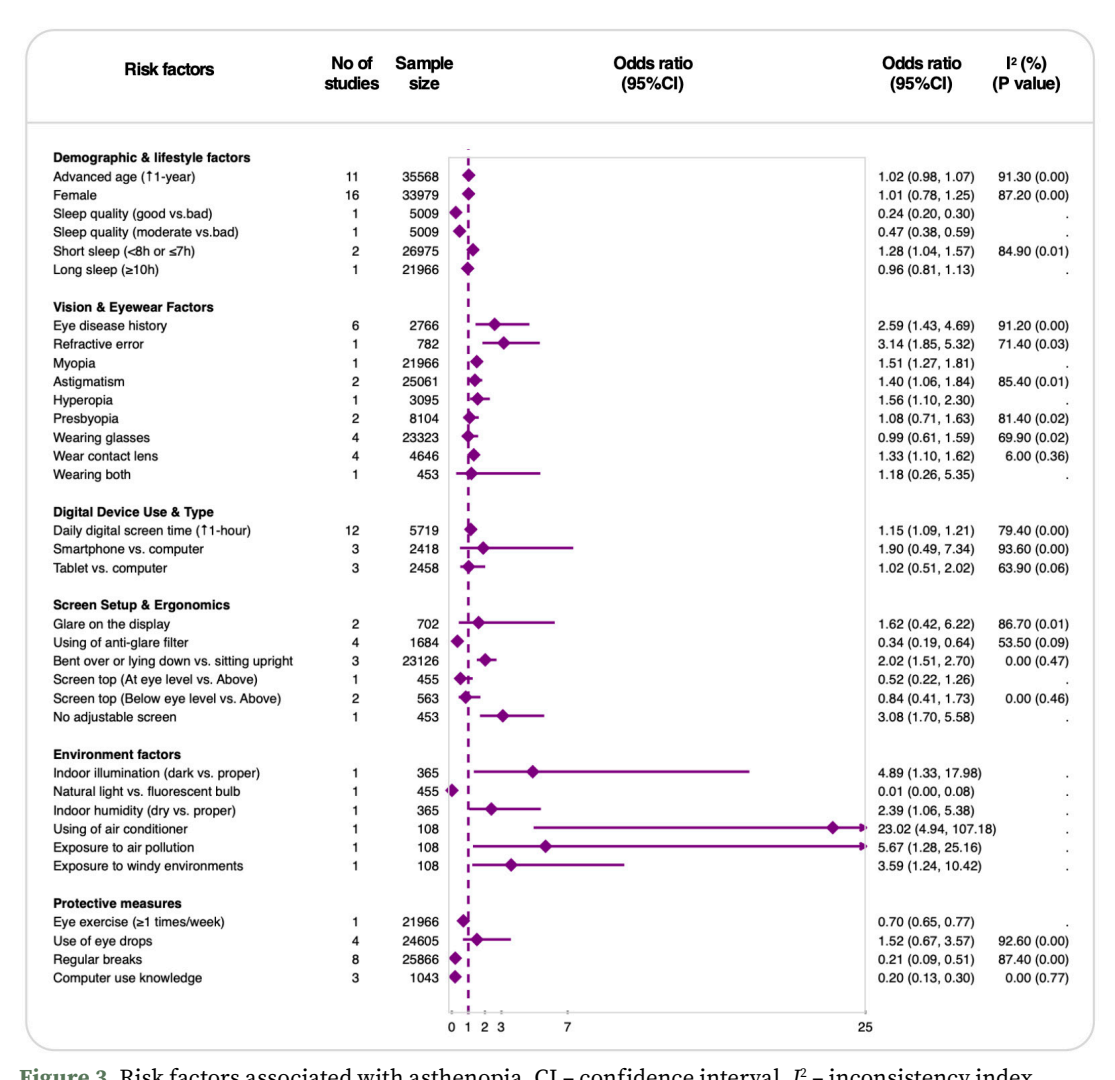
Risk factors associated with asthenopia. CI – confidence interval, *I*^2^ – inconsistency index (measure of between-study heterogeneity).

After pooled analysis, several factors showed no significant effect on asthenopia, including advanced age per additional year (OR = 1.02; 95% CI = 0.98, 1.07; 11 studies; 35 568 participants), female gender (OR = 1.01; 95% CI = 0.78, 1.25; 16 studies; 33 979 participants), long sleep duration over 10 hours (OR = 0.96; 95% CI = 0.81, 1.13; one study; 21 966 participants), presbyopia (OR = 1.08; 95% CI = 0.71, 1.63; two studies; 8104 participants), wearing glasses (OR = 0.99; 95% CI = 0.61, 1.59; four studies; 23 323 participants), type of digital device used (OR = 1.90; 95% CI = 0.49, 7.34; three studies; 2418 participants), screen position at eye level (OR = 0.84; 95% CI = 0.41, 1.73; two studies; 563 participants), and use of eye drops (OR = 1.52; 95% CI = 0.67, 3.57; four studies; 24 605 participants). The findings are summarised in **Figure 3**.

In sensitivity analyses restricted to studies with low or moderate risk of bias, ORs for most risk and protective factors were very similar to those in the main analysis. For some risk factors, the ORs changed, such as astigmatism (sensitivity analysis: OR = 1.20; 95% CI = 0.96, 1.40 *vs*. primary analysis: OR = 1.40; 95% CI = 1.06, 1.84) and glare on the display (OR = 3.30; 95% CI = 1.51, 7.21 *vs*. OR = 1.62; 95% CI = 0.42, 6.22). Some environmental factors (*e.g*. use of an air conditioner) showed high ORs but were based on one or two small studies with wide CIs, so these findings should be interpreted with caution (Figure S5 in the **Online Supplementary Document**).

## DISCUSSION

In this systematic review and meta-analysis, we evaluated the common clinical manifestations of asthenopia, its overall prevalence and sub-group prevalence across diverse populations, and risk factors. A total of 63 studies involving 60 589 participants from 25 countries were included. The majority of studies were conducted in India and several Arab countries. The school-aged children attending online classes during the COVID-19 pandemic were the most frequently studied population. The convergence of ocular, musculoskeletal, and neuropsychological symptoms, particularly eye tiredness (65%), neck pain (45%), and headache (50%), illustrates the systemic burden of asthenopia. Overall, more than half of the participants experienced asthenopia, although substantial heterogeneity was observed across studies. Importantly, several modifiable risk factors were identified, including prolonged screen time, poor ergonomics, and suboptimal ambient conditions such as poor lighting and low humidity. In contrast, protective behaviours such as taking regular breaks and performing eye exercises were associated with a reduced risk of asthenopia. These findings highlight the growing public health relevance of asthenopia in the digital era and underscore the need for targeted prevention and intervention. Part of this burden likely reflects COVID-era conditions and may not generalise to current settings. Accordingly, COVID-period estimates may be interpreted as context-specific. In addition, some risk factors (*e.g*. use of air conditioner) showed high odds ratios but were based on one or two small studies with wide confidence intervals; these findings should be interpreted with caution.

### Comparison with existing evidence

This systematic review and meta-analysis present the most comprehensive synthesis to date on asthenopia, combining prevalence estimates across diverse populations with an analysis of its clinical manifestations and risk factors. To our knowledge, this is the first systematic review to systematically categorise and quantify the full spectrum of clinical symptoms associated with asthenopia. Although many studies have reported symptoms of asthenopia, diagnostic criteria vary considerably, and no single validated questionnaire currently encompasses all reported symptoms. A side-by-side comparison with recent key systematic reviews is provided in Table S4 in the **Online Supplementary Document**. To address this, we conducted a meta-analysis of clinical manifestations reported across studies and grouped the symptoms into four categories: ocular symptoms, musculoskeletal symptoms, neuropsychological symptoms, and other symptoms. In the absence of a universally accepted symptom framework for asthenopia, this categorisation may serve as a foundation for future diagnostic criteria applicable across populations.

While several previous meta-analyses have examined the prevalence of asthenopia in specific groups such as university students, children, workers, or the general population, none have combined these aspects within a single review or investigated symptom profiles and contributing factors in such depth [1,3]. Our findings therefore extend existing evidence by providing a more comprehensive and multidimensional view of asthenopia across diverse demographic and occupational settings. A 2023 systematic review [3] reported a prevalence of computer vision syndrome (CVS) of 69%, whereas our analysis yielded an overall prevalence of 51%, both with considerable heterogeneity. After excluding studies with a high risk of bias, our adjusted prevalence estimate increased to 60%, aligning more closely with previous findings. This heterogeneity likely reflects variations in study populations, risk exposures, and inclusion criteria, making it a common limitation in asthenopia-related meta-analyses.

Another systematic review of young screen users identified poor ergonomic practices, lack of ocular rest, and prolonged screen time as significant risk factors for digital eye strain (DES), which is consistent with our findings [2]. Compared with that review, our analysis included a wider range of populations and study settings. We additionally identified lifestyle factors and eye conditions, such as short sleep duration, history of eye disease, hyperopia, myopia, and astigmatism, as being associated with an increased risk of asthenopia.

### Recommended definition of asthenopia

Asthenopia currently lacks a universally accepted definition, complicating both diagnosis and management. Based on our systematic review and meta-analysis, we propose defining asthenopia as a multidimensional syndrome characterised by a constellation of ocular, musculoskeletal, and neuropsychological symptoms arising primarily from prolonged near-visual tasks, especially in digital environments.

Our analysis identified 18 key symptoms reported by at least 30% of affected individuals, including eye tiredness, strain, burning or irritation, itching, light sensitivity, eye pain, visual deterioration, dryness, difficulty focusing or reading, blurred vision, lacrimation, redness, excessive blinking, neck pain, shoulder pain, headache, sleep or memory disturbances, and difficulty concentrating. Furthermore, key risk factors associated with asthenopia include insufficient sleep, prolonged digital screen time, poor screen ergonomics, eye disease history, refractive error, contact lens wear, and unfavourable ambient conditions (*e.g*. inadequate lighting, low humidity).

As a provisional framework to harmonise reporting and support pilot clinical use, we recommend that asthenopia be diagnosed when the following criteria are met:

1) Core symptom requirement: presence of at least one core symptom (*e.g*. eye tiredness, strain, neck pain, headache, or difficulty concentrating) during or within 30 minutes after near-work activities, after consideration of alternative ocular or systemic causes in line with the study or clinical settings.

2) Risk factor corroboration: documentation of at least one clinically risk factor, including but not limited to: prolonged digital device exposure (>4 hours/d), poor screen ergonomics (*e.g*. lack of an adjustable screen, improper sitting posture), or inadequate ambient lighting.

This case definition is intended for reporting and pilot screening; clinical judgment remains essential, and thresholds (*e.g*. symptom number/severity) require validation to minimise overdiagnosis.

We advocate a stepwise approach: piloting and validating this framework across diverse settings, including resource-limited settings; if supported, standardised adoption could improve diagnostic reproducibility and enable earlier identification through systematic screening.

### Relevance for clinical practice and research

The findings of this review have important implications for both clinical practice and public health. Clinically, our proposed multidimensional definition and symptom categories provide a clearer framework to improve diagnostic consistency for asthenopia and can support symptom screening in schools and workplaces using validated tools (*e.g*. CVS-Q, ASQ-11), which can also inform national guidance. Awareness of modifiable risk factors (screen time, ergonomics, ambient conditions) enables brief preventive counselling and intervention, with the aim of reducing symptom burden and improving quality of life. In practice, guidance can emphasise scheduled breaks (*e.g*. the 20–20–20 rule), reasonable screen-time limits, glare control or anti-glare filters, adequate lighting and humidity, and simple ergonomics, including upright sitting and an adjustable monitor. Regular eye exercises may also be encouraged where appropriate.

For researchers, this study underscores the need for standardisation in diagnostic criteria and measurement tools. Future research should prioritise the development and validation of subjective questionnaires that fully capture the spectrum of asthenopia symptoms. Concurrently, objective indicators such as eye movement parameters warrant further investigation to improve diagnostic accuracy and minimise bias. Longitudinal studies are also needed to explore causal relationships and evaluate the effectiveness of interventions. Additionally, more high-quality studies from less-studied regions are essential to enhance the global generalisability of findings.

### Strengths and limitations of this review

This systematic review has several notable strengths. First, it is the most comprehensive synthesis to date on asthenopia, incorporating data from a wide range of populations, study designs, and geographical settings. By integrating prevalence estimates, symptom profiles, and risk factors, this review provides a multidimensional perspective that advances understanding beyond the scope of previous subgroup-specific meta-analyses. Second, our systematic categorisation and meta-analysis of clinical manifestations offers a novel framework that may inform future diagnostic criteria, especially in the absence of a universally accepted definition of asthenopia.

However, several limitations should be acknowledged. First, although no language restrictions were applied during the search process, the final selection predominantly included studies published in English. This may have introduced language bias, as relevant studies in other languages may have been excluded due to accessibility and feasibility constraints. Second, considerable heterogeneity across studies was evident, driven by a range of factors such as diagnostic methods (*e.g*. CVS-Q vs symptom-based assessments), population characteristics (*e.g*. occupational exposure, age), and study settings. Although random-effects models were used to account for this variability, comparability across studies remains limited. Third, reliance on self-reported symptoms without objective measures may introduce potential recall and reporting biases, reducing diagnostic accuracy and validity. Future studies should include objective measures to improve reliability and comparability. Fourth, we included only recent studies to reflect current practice; estimates are therefore contemporary and may omit earlier foundational work. Reference lists were screened for widely used instruments. Fifth, cross-regional differences in work environments, screen-use habits, and health literacy may limit transportability, and most included studies were from India and several Arab countries, which may limit global generalisability. Moreover, key potential confounders were inconsistently measured or not reported across studies; thus residual confounding and limited comparability are possible. Therefore, application of our estimates and the proposed framework should be locally adapted. Lastly, some findings, especially those related to environmental factors, were based on limited evidence and should be interpreted with caution and explored in future studies.

## CONCLUSIONS

In summary, this study provides the most comprehensive evaluation of asthenopia to date, revealing its diverse clinical manifestations, high prevalence, and risk factors across multiple populations. By identifying key symptoms and key risk factors, our study proposes a unified definition and improved diagnostic criteria on asthenopia. Adoption of these findings in clinical practice may facilitate earlier detection and more effective management of asthenopia, ultimately reducing its burden in the digital era.

## Additional material


Online Supplementary Document
