# Supplementary material for: Clinical manifestations, prevalence, and risk factors of asthenopia: a systematic review and meta-analysis
Source: J Glob Health. 2026 Feb 6;16:04053. doi: 10.7189/jogh.16.04053 (PMC12879263; doi:10.7189/jogh.16.04053)

Supplement to: Song F, Liu Y, Zhao Z, Shang X, Wang Y, Lai M, He M, Chen Y. Clinical manifestations, prevalence, and risk factors of asthenopia: a systematic review and meta-analysis. J Glob Health. 2026;16:04053.

Table S1. PRISMA 2020 checklist.
Table S2. Electronic search strategy for PubMed.
Table S3. Quality assessment of included studies in the systematic review using the adapted Newcastle-Ottawa Scale (NOS).
Table S4. Comparison of key systematic reviews and meta-analyses on asthenopia (2015–2024).

Figure S1. PRISMA flow diagram of the study selection process.
Figure S2. Sensitivity analysis of asthenopia prevalence: restriction to studies with low or moderate risk of bias.
Figure S3. Sensitivity analysis of pooled asthenopia prevalence using the logit transformation.
Figure S4. Sensitivity analysis of asthenopia prevalence: restriction to studies using validated questionnaires.
Figure S5. Sensitivity analysis of risk factors: restriction to studies with low or moderate risk of bias.

Table S1. PRISMA 2020 checklist

Section and Topic	Item #	Checklist item	Location where item is reported
**TITLE**			
Title	1	Identify the report as a systematic review.	1
**ABSTRACT**			
Abstract	2	See the PRISMA 2020 for Abstracts checklist.	1-2
**INTRODUCTION**			
Rationale	3	Describe the rationale for the review in the context of existing knowledge.	2-3
Objectives	4	Provide an explicit statement of the objective(s) or question(s) the review addresses.	3
**METHODS**			
Eligibility criteria	5	Specify the inclusion and exclusion criteria for the review and how studies were grouped for the syntheses.	3
Information sources	6	Specify all databases, registers, websites, organisations, reference lists and other sources searched or consulted to identify studies. Specify the date when each source was last searched or consulted.	3-4
Search strategy	7	Present the full search strategies for all databases, registers and websites, including any filters and limits used.	4
Selection process	8	Specify the methods used to decide whether a study met the inclusion criteria of the review, including how many reviewers screened each record and each report retrieved, whether they worked independently, and if applicable, details of automation tools used in the process.	4
Data collection process	9	Specify the methods used to collect data from reports, including how many reviewers collected data from each report, whether they worked independently, any processes for obtaining or confirming data from study investigators, and if applicable, details of automation tools used in the process.	4
Data items	10a	List and define all outcomes for which data were sought. Specify whether all results that were compatible with each outcome domain in each	4

Section and Topic	Item #	Checklist item	Location where item is reported
		study were sought (e.g. for all measures, time points, analyses), and if not, the methods used to decide which results to collect.	
	10b	List and define all other variables for which data were sought (e.g. participant and intervention characteristics, funding sources). Describe any assumptions made about any missing or unclear information.	4
Study risk of bias assessment	11	Specify the methods used to assess risk of bias in the included studies, including details of the tool(s) used, how many reviewers assessed each study and whether they worked independently, and if applicable, details of automation tools used in the process.	4
Effect measures	12	Specify for each outcome the effect measure(s) (e.g. risk ratio, mean difference) used in the synthesis or presentation of results.	5
Synthesis methods	13a	Describe the processes used to decide which studies were eligible for each synthesis (e.g. tabulating the study intervention characteristics and comparing against the planned groups for each synthesis (item #5)).	4
	13b	Describe any methods required to prepare the data for presentation or synthesis, such as handling of missing summary statistics, or data conversions.	5
	13c	Describe any methods used to tabulate or visually display results of individual studies and syntheses.	5
	13d	Describe any methods used to synthesize results and provide a rationale for the choice(s). If meta-analysis was performed, describe the model(s), method(s) to identify the presence and extent of statistical heterogeneity, and software package(s) used.	5
	13e	Describe any methods used to explore possible causes of heterogeneity among study results (e.g. subgroup analysis, meta-regression).	5
	13f	Describe any sensitivity analyses conducted to assess robustness of the synthesized results.	5
Reporting bias assessment	14	Describe any methods used to assess risk of bias due to missing results in a synthesis (arising from reporting biases).	4
Certainty assessment	15	Describe any methods used to assess certainty (or confidence) in the body of evidence for an outcome.	4
**RESULTS**			

Section and Topic	Item #	Checklist item	Location where item is reported
Study selection	16a	Describe the results of the search and selection process, from the number of records identified in the search to the number of studies included in the review, ideally using a flow diagram.	5
	16b	Cite studies that might appear to meet the inclusion criteria, but which were excluded, and explain why they were excluded.	5
Study characteristics	17	Cite each included study and present its characteristics.	6-7
Risk of bias in studies	18	Present assessments of risk of bias for each included study.	6
Results of individual studies	19	For all outcomes, present, for each study: (a) summary statistics for each group (where appropriate) and (b) an effect estimate and its precision (e.g. confidence/credible interval), ideally using structured tables or plots.	6-11
Results of syntheses	20a	For each synthesis, briefly summarise the characteristics and risk of bias among contributing studies.	6-11
	20b	Present results of all statistical syntheses conducted. If meta-analysis was done, present for each the summary estimate and its precision (e.g. confidence/credible interval) and measures of statistical heterogeneity. If comparing groups, describe the direction of the effect.	6-11
	20c	Present results of all investigations of possible causes of heterogeneity among study results.	9-10
	20d	Present results of all sensitivity analyses conducted to assess the robustness of the synthesized results.	8, 11
Reporting biases	21	Present assessments of risk of bias due to missing results (arising from reporting biases) for each synthesis assessed.	N/A
Certainty of evidence	22	Present assessments of certainty (or confidence) in the body of evidence for each outcome assessed.	6
**DISCUSSION**			
Discussion	23a	Provide a general interpretation of the results in the context of other evidence.	11-12

Section and Topic	Item #	Checklist item	Location where item is reported
	23b	Discuss any limitations of the evidence included in the review.	15-16
	23c	Discuss any limitations of the review processes used.	15-16
	23d	Discuss implications of the results for practice, policy, and future research.	14
**OTHER INFORMATION**			
Registration and protocol	24a	Provide registration information for the review, including register name and registration number, or state that the review was not registered.	3
	24b	Indicate where the review protocol can be accessed, or state that a protocol was not prepared.	3
	24c	Describe and explain any amendments to information provided at registration or in the protocol.	N/A
Support	25	Describe sources of financial or non-financial support for the review, and the role of the funders or sponsors in the review.	16
Competing interests	26	Declare any competing interests of review authors.	16
Availability of data, code and other materials	27	Report which of the following are publicly available and where they can be found: template data collection forms; data extracted from included studies; data used for all analyses; analytic code; any other materials used in the review.	N/A

Table S2. Electronic search strategy for PubMed.

Search	Search query*
#1	"Asthenopia"[MeSH Terms]
#2	"fatigue"[Title/Abstract] OR "strain"[Title/Abstract]
#3	"eye"[Title/Abstract] OR "ocular"[Title/Abstract] OR "visual"[Title/Abstract]
#4	#1 OR (#2 AND #3)

MeSH - Medical Subject Headings. *The search was performed using the keywords search strategy.The search was performed using the keywords search strategy.

Table S3. Quality assessment of included studies in the systematic review using the adapted Newcastle-Ottawa Scale (NOS).

Quality assessment of included studies in the systematic review using the adapted NOS assessment scale										
Study	Selection						Outcome			Overall total
	Representativeness of the cases	Sample size	Non-Response rate	Ascertainment of the screening/surveillance tool	Subtotal	Comparability	Assessment of the outcome	Statistical test	Subtotal	Total/10	Summary
Abed Alah 2023	1	0	1	2	4	2	2	1	3	9	High
Almalki 2023	1	1	1	2	5	2	2	1	3	10	High
Almudhaiyan 2023	1	0	0	2	3	2	2	1	3	8	High
Bahkir 2020	1	1	1	1	4	0	1	1	2	6	Moderate
Basnet 2022	0	1	1	1	3	0	1	1	2	5	Moderate
Bhatnagar 2023	1	0	0	1	2	0	1	1	2	4	Low
Cantó-Sancho 2022	1	1	1	2	5	0	2	1	3	8	High
Chattinnakorn 2023	1	0	1	2	4	2	2	1	3	9	High
Gadain 2023	1	1	1	2	5	2	2	1	3	10	High
Galindo-Romero 2023	1	0	0	2	3	0	2	1	3	6	Moderate
Gammoh 2021	1	1	1	2	5	0	2	1	3	8	High
Mohan 2020	1	0	1	2	4	2	2	1	3	9	High
Moldovan 2019	1	0	1	1	3	2	1	1	2	7	High
Munsamy 2022	1	0	0	2	3	2	2	1	3	8	High
Nunes 2023	1	0	0	2	3	0	2	1	3	6	Moderate
Patel 2023	1	1	1	2	5	0	2	1	3	8	High
Poudel 2020	1	1	1	0	3	2	0	1	1	6	Moderate

Sengo 2023	0	1	1	2	4	2	2	1	3	9	High
Shah 2021	1	0	0	1	2	0	1	1	2	4	Low
Al Dandan 2020	1	0	1	1	3	2	1	1	2	7	High
Balsam Alabdulkader 2021	1	1	1	1	4	2	1	1	2	8	High
Abdulrahman Alamri 2023	0	1	1	1	3	1	1	1	2	6	Moderate
Abdulrhman Aldukhayel 2022	0	1	1	2	4	2	2	1	3	9	High
Hassan M. Alturaiki 2023	1	0	0	2	3	1	2	1	3	7	High
Saif K. Dossari 2022	1	1	0	1	3	1	1	1	2	6	Moderate
Mohammad Abusamak 2022	0	1	1	1	3	2	1	1	2	7	High
Johanna Coronel-Ocampos 2022	0	1	1	2	4	2	2	1	3	9	High
Sourav Datt 2023	0	0	1	2	3	0	2	1	3	6	Moderate
Bengi Demirayak 2022	1	1	1	1	4	2	1	1	2	8	High
Samuel Bert Boadi-Kusi 2021	0	0	1	2	3	1	2	1	3	7	High
Richa Agarwal	1	0	0	1	2	1	1	1	2	5	Moderate
Abdulrahman AlDarrab 2021	1	1	1	1	4	1	1	1	2	7	High
Dora Hamad AlHarkan 2023	1	1	1	2	5	1	2	1	3	9	High

Hira Nath Dahal 2022	1	0	1	1	3	0	1	1	2	5	Moderate
Muhammad Junaid Tahir 2024	1	1	1	1	4	2	1	1	2	8	High
Hyojin Kim 2024	1	1	1	1	4	2	1	1	2	8	High
Catherine Wang 2023	1	0	1	2	4	0	2	1	3	7	High
Priyanka Shrestha 2023	1	1	1	1	4	0	1	1	2	6	Moderate
Aishwarya Sharma 2023	1	1	1	2	5	2	2	1	3	10	High
Na Lin 2023	1	1	1	2	5	2	2	1	3	10	High
Fiza Jakhar 2023	1	0	1	1	3	2	1	1	2	7	High
Sonia Celedonia Huyhua-Gutierrez 2023	1	1	1	2	5	2	2	1	3	10	High
Marwa M Zalat 2022	1	1	0	2	4	0	2	1	3	7	High
Kampanat Wangsan 2022	1	1	1	2	5	1	2	1	3	9	High
Alexandre Uwimana 2022	1	0	1	2	4	2	2	1	3	9	High
Cristian Talens-Estarelles 2022	1	1	1	2	5	2	2	1	3	10	High
Ioanna Mylona 2022	1	1	0	2	4	1	2	1	3	8	High
Nancy M Lotfy 2022	0	0	1	1	2	2	1	1	2	6	Moderate
Kirandeep Kaur 2021	0	1	1	0	2	0	0	1	1	3	Low
Fuhao Zheng 2021	0	0	0	1	1	0	1	1	2	3	Low

Hanaa Abdelaziz Mohamed Zayed 2021	1	1	0	2	4	2	2	1	3	9	High
Lin Li 2021	1	0	0	2	3	2	2	1	3	8	High
Richa Gupta 2021	0	1	1	2	4	0	2	1	3	7	High
Pratyusha Ganne 2021	1	0	0	2	3	1	2	1	3	7	High
Rayah Issam Touma Sawaya 2020	1	0	0	1	2	2	1	1	2	6	Moderate
Mahlet Getachew Lemma 2020	1	1	1	1	4	2	1	1	2	8	High
Barbara M. Junghans 2020	1	0	1	2	4	0	2	1	3	7	High
Caleb Teo 2019	0	0	0	2	2	0	2	1	3	5	Moderate
Chu 2023	1	0	1	2	4	2	2	1	3	9	High
Y Ding 2023	1	0	0	1	2	2	1	1	2	6	Moderate
Francesca Larese Filon 2019	1	1	0	1	3	2	1	1	2	7	High
Asakawa 2020	1	0	0	1	2	0	1	1	2	4	Low
Rémi Coq 2024	0	0	1	2	3	0	2	1	3	6	Moderate

Table S4. Comparison of Key Systematic Reviews and Meta-Analyses on Asthenopia (2015–2024)

First Author (Year)	Core Terminology	Population Scope	Geographical Scope / Region(s)	Search Period	Studies Included (Participants)	Diagnostic Instruments	Primary Outcome(s)	Key Estimate(s) / Finding(s)
Darwin A León-Figueroa (2024)	CVS, DES	General population (students & non-students) during COVID-19	Global	Until Feb 2023	18 (10,337)	Primarily subjective questionnaires & or symptom checklists	Prevalence	Pooled prevalence: 74% (95% CI: 66–81). Higher in non-students (82%) than students (70%).
Fabricio Ccami-Bernal (2023)	Computer Vision Syndrome (CVS)	General population (no age/occupation restrictions)	Global	Until Feb 2023	103 (66,577)	Validated (e.g., CVS-Q) & non-validated questionnaires (symptom checklists)	Prevalence	Pooled prevalence: 69.0% (95% CI: 62.3%–75.3). Higher in university students (76.1%), women (71.4%) and and in those that did not use the CVS-Q questionnaire (75.4%).
Etsay Woldu Anbesu (2023)	CVS, Digital Eye Strain (DES), Asthenopia	General population	Global	No restriction	45 (17,526)	Primarily subjective questionnaires & presence of ≥1 symptom	Prevalence	The pooled prevalence of computer vision syndrome was 66% (95% CI: 59, 74). Subgroup analysis based on country was highest in Pakistan (97%,

								95% CI: 96, 98) and lowest in Japan (12%, 95% CI: 9, 15).
Asamene Kelelom Lema (2022)	CVS, DES, visual fatigue	General population	Global	No restriction	49 (23,399)	Primarily subjective questionnaires	Prevalence & Determinants (associated factors) of CVS	The pooled prevalence of computer vision syndrome (CVS) was 66%. Key risk factors were: being female (OR=1.74), improper posture (OR=2.65), using devices after work (OR=1.66), not taking breaks (OR=2.24), long screen time (OR=2.02), short viewing distance (OR=4.24), and poor ergonomics (OR=3.87). Good knowledge about CVS was protective (OR=4.04).
Manuel A P Vilela (2015)	Asthenopia, eye fatigue, visual fatigue, and eyestrain	Children (0–18 years)	Global	1960–May 2014	5 (2,465)	subjective symptom reports	Prevalence	Pooled prevalence: 19.7% (95% CI: 12.4%–26.4%). The prevalence in the single largest study (on 6-year-olds) was lower, at 12.6%.
Our study	Asthenopia, eye fatigue, visual fatigue, and eye strain	General population	Global	Studies published within the last five years up to 26 April 2024	63 (60,589)	Subjective questionnaires & or symptom checklists	Prevalence, clinical manifestations, and risk factors	Pooled Prevalence of Asthenopia: 51% (95% CI: 50%, 52%).<br>Key Clinical Manifestations:<br>- Ocular: Eye tiredness (65%), eye strain (47%), burning/irritation (43%)<br>- Musculoskeletal: Neck pain (45%), shoulder pain (30%)<br>- Neuropsychological: Headache (50%), difficulty concentrating (44%)

								Key Risk Factors:<br>- Increased Risk: Short sleep duration (OR=1.28), prior eye disease (OR=2.59), prolonged screen time (OR=1.15 per hour), air conditioning use (OR=23.02), improper sitting posture (OR=2.02).<br>- Protective Factors: Use of anti-glare filters (OR=0.34), taking regular breaks (OR=0.21), having computer-use knowledge (OR=0.20).

Figure S1. PRISMA flow diagram of study selection process.

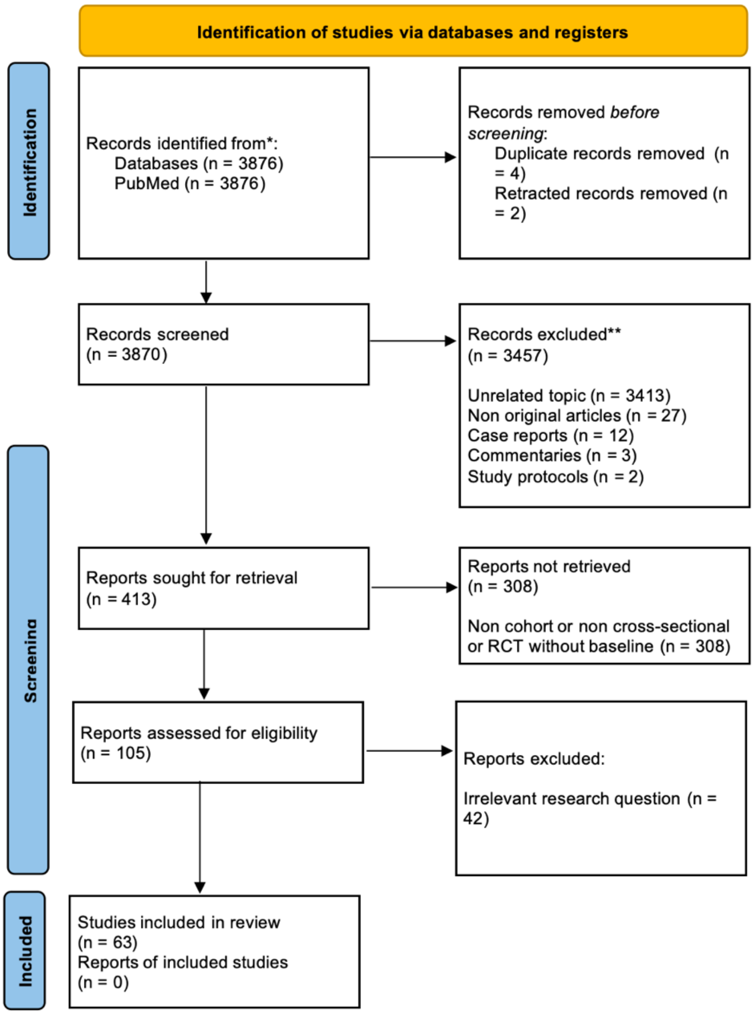

Figure S2. Sensitivity Analysis of Asthenopia Prevalence: Restriction to Studies with Low or Moderate Risk of Bias. CI – confidence interval; No of events – number of events; COVID-19 – coronavirus disease 2019; ASQ-11 – Asthenopia Survey Questionnaire-11; CISS – Convergence Insufficiency Symptom Survey; CVS-Q – Computer Vision Syndrome Questionnaire.

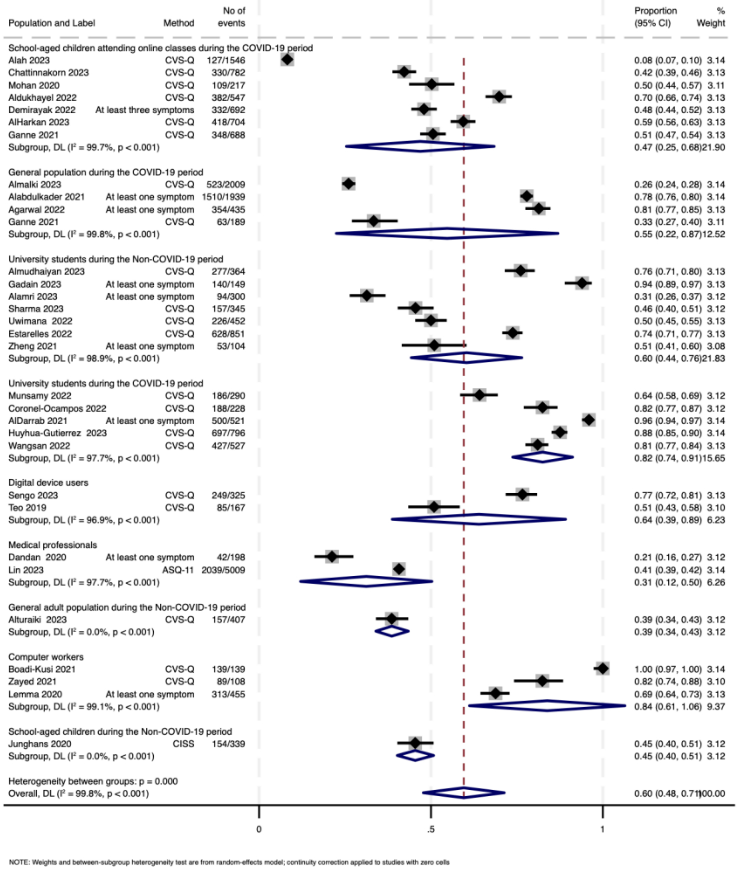

Figure S3. Sensitivity analysis of pooled asthenopia prevalence using the logit transformation. CI – confidence interval; I² – inconsistency index (measure of between-study heterogeneity); No of events – number of events; COVID-19 – coronavirus disease 2019; ASQ-11 – Asthenopia Survey Questionnaire-11; CISS – Convergence Insufficiency Symptom Survey; CVS-Q – Computer Vision Syndrome Questionnaire.

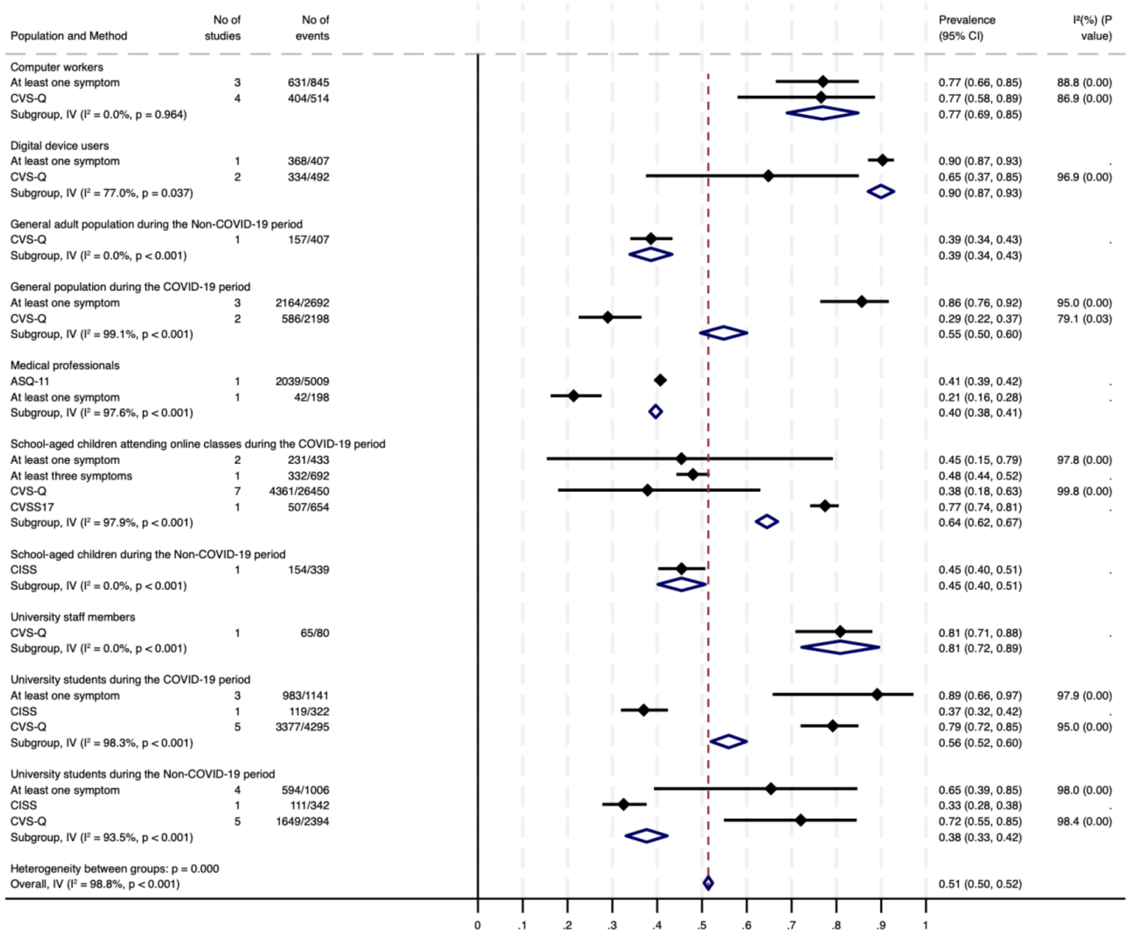

Figure S4. Sensitivity Analysis of Asthenopia Prevalence: Restriction to Studies using Validated questionnaires. CI – confidence interval; I^2 – inconsistency index (measure of between-study heterogeneity); No of events – number of events; COVID-19 – coronavirus disease 2019; ASQ-11 – Asthenopia Survey Questionnaire-11; CISS – Convergence Insufficiency Symptom Survey; CVS-Q – Computer Vision Syndrome Questionnaire.

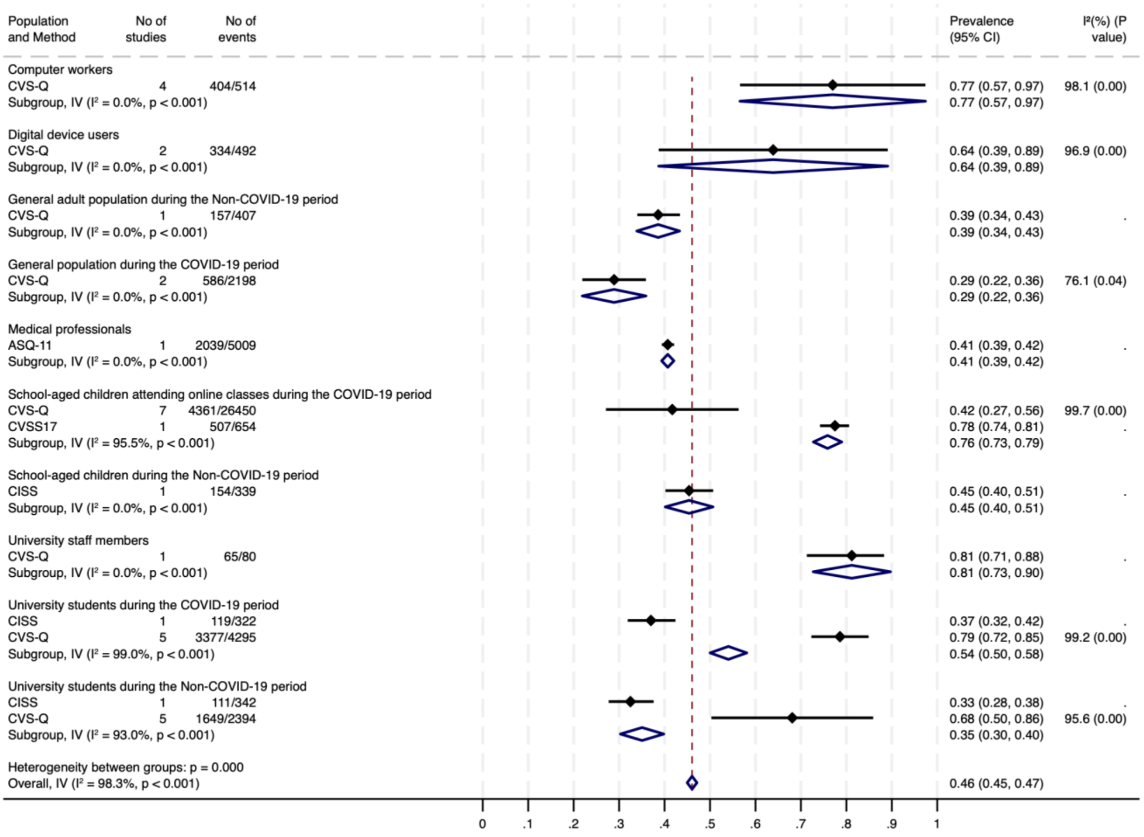

Figure S5. Sensitivity Analysis of Risk Factors: Restriction to Studies with Low or Moderate Risk of Bias. CI – confidence interval; I² – inconsistency index (measure of between-study heterogeneity); No of studies – number of included studies.

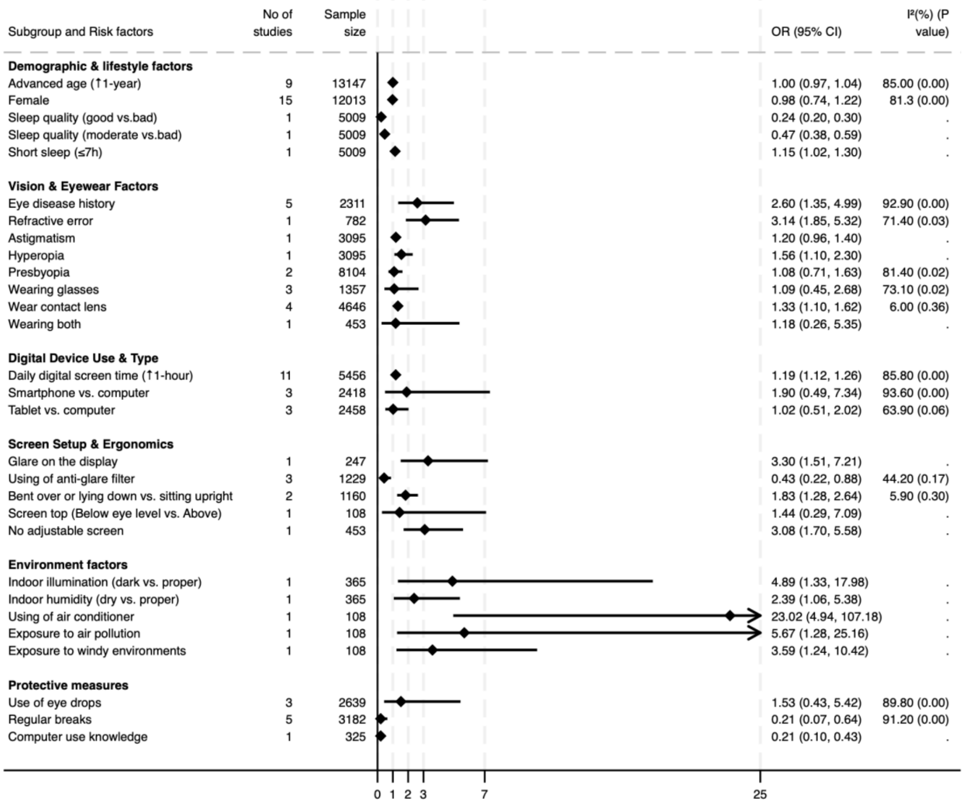

Supplement: Online Supplementary Document [file jogh-16-04053-s001.pdf]
